# Ecological and Anthropogenic Spatial Gradients Shape Patterns of Dispersal of Foot-and-Mouth Disease Virus in Uganda

**DOI:** 10.3390/pathogens11050524

**Published:** 2022-04-29

**Authors:** Anna Munsey, Frank Norbert Mwiine, Sylvester Ochwo, Lauro Velazquez-Salinas, Zaheer Ahmed, Luis L. Rodriguez, Elizabeth Rieder, Andres Perez, Kimberly VanderWaal

**Affiliations:** 1 Veterinary Population Medicine Department, University of Minnesota College of Veterinary Medicine, St. Paul, MN 55108, USA; aperez@umn.edu (A.P.); kvw@umn.edu (K.V.); 2 College of Veterinary Medicine, Animal Resources and Biosecurity (COVAB), Makerere University, Kampala 7072, Uganda; fmwiine@gmail.com (F.N.M.); ochwosylver@gmail.com (S.O.); 3 Foreign Animal Disease Research Unit, Plum Island Animal Disease Center, Agricultural Research Service (ARS), United States Department of Agriculture, Greenport, NY 11957, USA; lauro.velazquez@usda.gov (L.V.-S.); zaheer.ahmed@usda.gov (Z.A.); luis.rodriguez@usda.gov (L.L.R.); elizabeth.rieder@usda.gov (E.R.)

**Keywords:** livestock markets, molecular epidemiology, regression models, spatial analysis, disease ecology

## Abstract

Using georeferenced phylogenetic trees, phylogeography allows researchers to elucidate interactions between environmental heterogeneities and patterns of infectious disease spread. Concordant with the increasing availability of pathogen genetic sequence data, there is a growing need for tools to test epidemiological hypotheses in this field. In this study, we apply tools traditionally used in ecology to elucidate the epidemiology of foot-and-mouth disease virus (FMDV) in Uganda. We analyze FMDV serotype O genetic sequences and their corresponding spatiotemporal metadata from a cross-sectional study of cattle. We apply step selection function (SSF) models, typically used to study wildlife habitat selection, to viral phylogenies to show that FMDV is more likely to be found in areas of low rainfall. Next, we use a novel approach, a resource gradient function (RGF) model, to elucidate characteristics of viral source and sink areas. An RGF model applied to our data reveals that areas of high cattle density and areas near livestock markets may serve as sources of FMDV dissemination in Uganda, and areas of low rainfall serve as viral sinks that experience frequent reintroductions. Our results may help to inform risk-based FMDV control strategies in Uganda. More broadly, these tools advance the phylogenetic toolkit, as they may help to uncover patterns of spread of other organisms for which genetic sequences and corresponding spatiotemporal metadata exist.

## 1. Introduction

Owing to the increasing availability of genetic sequence data, phylogenetic techniques have become a fundamental component in the study of the epidemiology of infectious diseases. Phylogeographic tools enable the reconstruction of the spatiotemporal dispersal history of pathogens [1], such that time-stamped phylogenetic trees can be visualized in a geographic context. Landscape epidemiology elucidates interactions between environmental heterogeneities and patterns of infectious disease spread using georeferenced phylogenetic trees [2]. For example, using phylogeographic approaches, Dellicour et al. demonstrated that West Nile virus lineages tend to disperse faster in areas with higher temperatures [3], and Zhang et al. demonstrated an association between the spatial spread of Middle East respiratory syndrome coronavirus and the regional transportation network [4]. Phylogeographic techniques can provide insights into the complex interplay between the pathogen, host, and environment and help to guide disease control strategies.

Here, we utilize foot-and-mouth disease virus (FMDV) sequences from Uganda as a case study for the development of novel tools in landscape epidemiology. While foot-and-mouth disease (FMD) has been eradicated from most developed countries, it remains widespread in Africa, where frequent outbreaks contribute to the cycle of poverty among livestock owners [5,6]. Infection with FMDV causes vesicular lesions in cloven-hooved animals, affecting animal welfare and causing short and long-term production losses [7]. FMDV is transmitted primarily through direct contact via respiratory droplets, but may also be transmitted by fomites [8]. An RNA virus in the *Picornaviridae* family, FMDV is characterized by a high nucleotide substitution rate. With seven immunologically distinct serotypes, as well as an extensive intra-serotypic diversity, the genetic diversity of FMDV presents a significant obstacle to control. However, this genetic diversity can be used to better understand disparities in risk across space, as its evolutionary dynamics occur on the same time scale as ecological and epidemiological dynamics.

A cross-sectional serological study carried out in Uganda found that the risk of FMDV exposure was not uniform across the country, with areas near international borders, particularly southwestern and eastern Uganda, having a relatively higher risk of FMDV exposure [9]. In addition, pastoral cattle herds, herds in areas of low rainfall, and herds in areas of high cattle density had relatively higher numbers of seropositive animals. The phylogeographic modeling of sequences obtained from the same cross-sectional study, combined with previous sequence data from Uganda, Kenya, Tanzania, and Ethiopia, re-affirmed the transboundary nature of the spatiotemporal spread of FMDV and identified a tendency for FMDV to remain circulating near livestock markets, in areas of high cattle density, and in areas of high human population density [10]. However, using available methods, no directional trends in FMDV spread were elucidated. Here, we sought to determine which external factors influence the dispersal direction of serotype O FMDV in Uganda, as the ability to predict directionality of FMDV spread may help to guide the application of limited resources (i.e., vaccination) in the face of an outbreak. Specifically, we aimed to (1) determine which environmental factors predict where, among available locations, FMDV is likely to disperse, and (2) characterize FMDV source and sink locations along a gradient of hypothesized environmental factors. In order to elucidate directional patterns in FMDV spread, we adapted a spatial regression tool that has historically been used in the wildlife ecology literature and developed a novel regression tool. Resource selection function (RSF) models were originally designed to correlate environmental covariates to animal location data by identifying characteristics of areas used by the animal within an “availability domain”, the area within which any location is assumed to be available for use [11,12]. In the context of wildlife ecology, these models are used to make inferences regarding habitat selection. More recently, step-selection function (SSF) models built upon RSF models by restricting the available, unobserved locations at time *t* to areas deemed accessible from the previous step at time *t* − 1 [13,14]. SSFs allow for the inference of movement processes in addition to habitat selection by including movement-related covariates, such as step lengths, and requiring that random, unobserved locations are generated by sampling under one of several statistical distributions [15,16,17,18]. In this first analysis, we apply the SSF framework to a phylogenetic model to determine which external factors are predictive of FMDV spatial spread given a defined availability domain.

In the second analysis, we developed a novel method, termed the resource gradient function (RGF) model, to identify patterns of FMDV source/sink dynamics. Source/sink models describe unequal gene flow: source regions serve as viral reservoirs from which outbreaks or epidemics tend to emerge, whereas sinks are areas in which the virus would be less likely to persist without repeated reintroduction from source regions. Thus, source regions are areas in which control efforts may have the greatest impact. The aim of the RGF model is to compare characteristics of locations where the branches originated to characteristics of locations where they ended, thus describing the resource gradient through which lineages tend to travel (i.e., from lower values to higher values, or vice versa), thereby identifying characteristics of FMDV source regions. Collectively, the SSF and RGF models serve to identify factors that contribute to the disproportionate transmission of pathogens. These methods could be applied to study the spread of any organism for which genetic sequences and corresponding spatiotemporal metadata exist.

## 2. Materials and Methods

### 2.1. Virus Sampling and Sequencing

FMDV viral sequences were collected as part of an FMDV surveillance study in Uganda previously described [19,20]. Serotype O was the most common serotype isolated; all isolates included in this analysis are serotype O, topotype EA-2. No additional serotype O sequences were available on GenBank during the time period analyzed here. Each cattle herd (*n* = 48) was sampled at only one point in time, resulting in high within-herd nucleotide identities. Thus, one sequence was randomly selected from each herd using R version 3.6.1. Sequences were aligned using ClustalW in MEGA (v10.0.5) [21].

### 2.2. Phylogeographic Analysis

First, the temporal signal of the data was evaluated in Tempest (v1.5) [22]. Using a linear regression of phylogenetic root-to-tip distances versus sampling dates, a positive correlation (R^2^ = 0.68) was demonstrated. jModeltest (2.1.10 v20160303) [23,24] was used to identify the best nucleotide substitution model. Next, we estimated FMDV spatial diffusion dynamics using a continuous phylogeographic method implemented in BEAST (v1.10.4) [25], in which, tree branches represent time and tree tips and internal nodes are associated with geographic locations. A Cauchy relaxed random walk (RRW) model was used for inference of the spatial locations of internal nodes.

Combinations of molecular clock models (uncorrelated lognormal relaxed, strict) and coalescent population models (constant, exponential, GMRF Bayesian skyride, logistic) were compared using path sampling/stepping-stone sampling [26,27], each using default priors. Results of path sampling/stepping-stone sampling are shown in Supplemental Table S1. Each molecular clock–population model combination was evaluated using the mean of the log marginal likelihood of two Markov chain Monte Carlo (MCMC) runs. Uncorrelated relaxed molecular clock models were run on the CIPRES Science Gateway (www.phylo.org, accessed on 2 January 2020) for 500 million generations, sampling every 50,000 generations; strict molecular clock models were run for 100 million generations, sampling every 10,000 generations. Tracer (v1.7.1) [28] was used to assess convergence of MCMC runs after excluding 10 percent of the MCMC chain as burn-in, ensuring an effective sample size (ESS) of at least 200 [29].

Next, the *seraphim* package in R (v 3.6.1) was utilized to extract the spatiotemporal information contained in the phylogenetic trees inferred in BEAST [30]. We extracted the information from 1000 trees randomly sampled from the posterior distribution after discarding burn-in. *seraphim* was then used to estimate dispersal statistics based on phylogenetic branches, which can be treated as conditionally independent movement vectors [31].

### 2.3. Directional Analyses

The output of *seraphim* was utilized such that each phylogenetic branch was considered a vector defined by a start and end location (latitude and longitude) and start and end dates. ArcGIS Pro 2.4.0 was used to calculate the vector direction (0–360) of branches between start and end locations. To test the significance of the mean direction of spread, a Rayleigh test was conducted using the R package circular [32]. R packages dplyr, ggtree, rBt, and treeio were used to integrate outputs from BEAST, *seraphim*, and ArcGIS analyses [33,34,35,36,37,38].

### 2.4. Step Selection Function Model

In order to investigate factors associated with the spatial spread of FMDV, we chose the following covariates as hypothesized predictors: mean annual rainfall, cattle density, human population density, Euclidean distance to nearest livestock market, Euclidean distance to nearest major roadway, and Euclidean distance to nearest international border. These factors were previously found to be associated with high seropositivity (proximity to international borders, low rainfall, and high cattle density); hence, we hypothesized that areas with such characteristics may serve as viral sources (i.e., frequently export the virus) [9]. Additionally, we predicted a tendency for FMDV to spread along routes of animal movement and trade—that is, a tendency for FMDV to spread toward roadways, livestock markets, and areas of high human population density. Sources of covariate data are shown in Supplemental Table S2.

In wildlife ecology, the movement from *t* − 1 to *t* is a “step”, and the distance traveled is the step length. We applied the step selection function framework to a phylogenetic model by treating individual branches of the phylogenetic tree as steps, and branch distance as step lengths. We then generated unobserved available locations (i.e., locations that the virus could have reached given spatio-temporal constraints on dispersal, but were not observed in our dataset) based on analytical distributions fitted to the inferred phylogenetic tree branch distances. We limited the analysis to branches with a duration of six months or less. The reasoning for this was two-fold: (1) to decrease the spatial uncertainty of the inferred node locations, and (2) to make predictions for an appropriate timeframe from an outbreak control standpoint. Among the sequences analyzed here, the mean dispersal velocity was 262.5 km (km)/year (95% HPD: 138.1–915.4); thus, we filtered our dataset to include only branches less than 131 km (i.e., mean distance traveled in 6 months) in length. This step eliminated the eight longest branches in the dataset, yielding a dataset of 86 branches. Branches in the full dataset had a median length of 8.4 km; the median branch length after subsetting to branches less than 131 km was 6.6 km.

**Figure 1:**
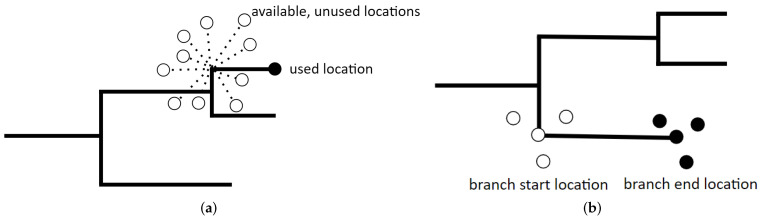
Diagrams depicting (**a**) the step selection function (SSF) model and (**b**) the resource gradient function (RGF) models used in this study. Both models have a binary response variable, depicted here by black and white circles. SSF models describe differences between used locations (black circles) and *n* = 10 available, unused locations per used location (white circles). RGF models describe differences between the areas where branches start locations (white circles) and where branches end location (black circles).

Next, we created a vector of FMDV movements from the output of the *seraphim* analysis. Using the amt package [39], one observed movement was generated from each branch of the phylogenetic tree, with each movement having a corresponding start location, end location, start date, and end date. Movement distance was calculated as the distance between start location and end location, which was treated as the step length for movement calculations. End locations of these movements are hereafter referred to as used locations, as these represent observed movements made by the virus. A gamma distribution was fit to the movement distances, and ten random distances were drawn from this distribution per observed movement (Figure 1a). Based on these random distances, unobserved end locations were generated to represent locations where the virus could have traveled (“availability domain”), but did not, under the same space–time constraints. Unobserved end locations are hereafter referred to as available locations. If prior information is available regarding the direction of viral spread, this can inform the turn angle used to generate the unobserved movements. In this case, however, we assumed the virus was equally likely to spread in any direction from the start location. Thus, a uniform distribution was used to generate the turn angle. The resulting available locations (*n* = 860) were filtered so that all remaining locations remained within Uganda’s boundaries. For example, if an available location was generated within Kenya or a body or water, it was removed. While we acknowledge that transboundary animal movement does occur, our dataset was artificially constrained to locations within Uganda (no comparable geo-referenced sequences were available from bordering countries); therefore, we built this same constraint into the matched, available locations. This step resulted in removal of 66 locations. Thus, our final dataset contained 86 used locations and 794 available locations. Next, covariate values at each end location (used and available) were extracted using amt [40]. A matrix showing correlation amongst predictors is shown in Supplemental Table S3; no significant correlations were identified. Covariates were categorized into terciles; cutoffs for the covariate categories are shown in Supplemental Table S4. Using the ResourceSelection package in R [40,41], SSF matched models were used to fit the end location data, with used/available as the binary response variable, using 99 bootstrap iterations. Backwards stepwise selection (Bayesian information criterion) was used to determine the best-fitting model.

### 2.5. Resource Gradient Function Model

The step selection function model compares characteristics of used relative to available branch-end locations without formally integrating the environmental characteristics of the start location. In contrast, the aim of the resource gradient function model is to compare characteristics of locations where the branches started to characteristics of locations where they ended, thus describing the resource gradient along which lineages tend to travel (i.e., from lower values to higher values, or vice versa). Whereas SSF models utilize an observed-available design, the RGF model utilizes a start-end design to elucidate differences between branch-start locations and branch-end locations. In this analysis, we generated three random neighboring locations per observed start location and end location, e.g., a “start domain” and “end domain” for each movement (Figure 1b). The inclusion of neighboring locations serves to account for uncertainty in the precise locations of inferred nodes by capturing the characteristics of the areas in close proximity to the branch start and end locations while simultaneously increasing the statistical power of the analysis. To accomplish this, the branch lengths were divided by four to avoid overlap in the start and end domains, then a gamma distribution of distances was generated from the resulting shorter branch lengths. Neighbor locations were randomly generated following the gamma distribution of shorter lengths. Neighbor locations were combined with the observed locations for the RGF analysis and the dataset was cropped to ensure all locations were within the study area. In this case, only one neighboring location was eliminated. Thus, the final dataset contained 86 start locations with 255 associated neighbor locations, and 86 end locations with 256 associated neighbor locations (total *n* = 683). Covariate values at each start and end location were extracted using amt [39], and the same covariates were tested as in the SSF; the predictor correlation matrix is shown in Supplemental Table S5. Covariates were again categorized into terciles; cutoffs for the covariate categories are shown in Supplemental Table S4. Locations less than 14.8 km from a livestock market were categorized as near a market; locations greater than 30.4 km were considered far from a market. The survival package was used to fit a matched conditional logistic regression model [42,43] with node type as the outcome of interest: branch-starts were coded as 1, branch-ends were coded as 0. Backwards stepwise selection (concordance statistic) was used to determine the best-fitting model.

### 2.6. Viral Source Map

In order to better understand FMDV source locations, we utilized the informative predictors in the RGF model (i.e., those that were retained in the best-fitting model) to generate a qualitative risk map. Rasters of informative covariates (*n* = 3) were centered, scaled, and inverted (where appropriate), such that high values represent relatively higher likelihood of being a source of FMDV (i.e., classified as a branch-start under the RGF model). A new raster was generated by calculating the per-cell mean of the rescaled covariate rasters, which was plotted using the raster package [44].

## 3. Results

### 3.1. Model Selection and Directional Statistics

Supplemental Figure S6 shows the location of Uganda within Africa; Supplemental Figure S7A shows the location of the collection of the 48 serotype O isolates used for this study. GenBank accession numbers are summarized in Supplemental Table S8. The best-fitting nucleotide substitution–molecular clock–population model was the Jukes–Cantor-strict clock–constant population combination [45]. The nucleotide substitution rate was 5.37 × 10${}^{-3}$ substitutions/site/year (95% HPD: 2.34 × 10${}^{-3}$− 8.55 × 10${}^{-3}$). The inferred phylogeny is shown in Supplemental Figure S9 and corresponding spatiotemporal diffusion is shown in Supplemental Figure S7B. For the time period assessed here, the overall direction of spread was 296.1 degrees, which is a west-northwest direction. The Raleigh test of directionality was not statistically significant; that is, the overall direction of spread was not significantly different from random.

### 3.2. Regression Models

**Figure 2:**
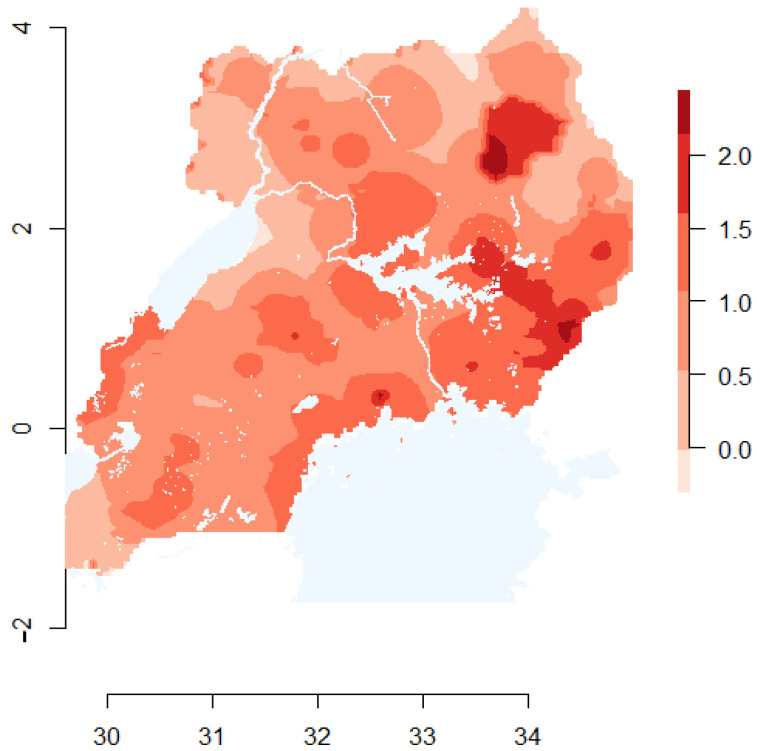
Map depicting the qualitative risk of being a source area of FMDV in Uganda. The shading represents the likelihood of serving as FMDV source areas, with darker shades representing higher risk. Data from informative covariates in the resource gradient function model (RGF) were centered, scaled, and inverted (where appropriate), such that high values represent a higher likelihood of being a source of FMDV (i.e., classified as a branch-start under the RGF model). The map depicts the mean of the rescaled predictors.

Under the step selection function model, the best fit model included only rainfall as a predictor. This finding was marginally significant (*p* = 0.056), with locations in the lowest category of annual rainfall having an odds ratio (OR) of 2.02 (95% confidence interval: 0.97–4.19). Thus, locations in areas of low rainfall were marginally more likely to be classified as a used location as opposed to an available (but unused) location. Under the resource gradient model, three predictors were informative: rainfall, cattle density, and distance to the nearest livestock market. RGF model ORs and 95% confidence intervals are shown in Table 1. Areas of high cattle density and in close proximity to livestock markets were more likely to be classified as a branch start location, indicating that they are more likely to be FMDV source areas. Areas of low cattle density and low annual rainfall were more likely to be classified as branch end locations, indicating that they are more likely to be FMDV sink areas. A map displaying the qualitative risk of FMDV source areas is shown in Figure 2.

## 4. Discussion

In this study, we have detailed two methods, the SSF model and the RGF model, for examining the spatial dynamics of viral phylogenies. These methods serve to advance the phylogenetic toolkit, as they may be used to study the spread of any organism for which genetic sequences and corresponding spatiotemporal metadata exist. We applied SSF and RGF models to identify factors that contribute to the disproportionate transmission of FMDV in Uganda. Under the SSF model, areas of low rainfall were marginally more likely to be classified as a used location. This finding is consistent with our earlier findings using serology data, which revealed a high seropositivity in low rainfall areas [9]. Drier conditions are expected to decrease the survivability of FMDV outside the host [46], so this pattern is not likely a result of the effect of rainfall on transmissibility. Rather, it is likely that rainfall serves as a proxy for factors not assessed here. For example, drier conditions alter the patterns of animal movement and resultant host contacts [47,48], as animals may move further for access to food and water and congregate around limited water resources. Additionally, dry conditions can result in poor body conditions, which may impact the host susceptibility to FMDV. The remaining covariates (cattle density, human population density, distance to roadway, distance to international border) were not informative in the SSF model.

In a previous study of FMDV phylogenetics using *seraphim*, we identified several characteristics of areas where FMDV is likely to remain circulating (near livestock markets, areas of high cattle density, and high human population density), but we were unable to elucidate directional patterns in FMDV spread [10]. Using the RGF model, we identified directional trends related to rainfall, cattle density, and proximity to livestock markets. Areas of low rainfall were more likely to be classified as branch-ends as opposed to branch-starts. The SSF model indicated that low rainfall areas were marginally more likely to be classified as used locations. Considered together, these findings provide evidence that areas of low rainfall can be considered sinks of FMDV serotype O in Uganda. That is, FMDV is frequently found in these areas, but, since they have low odds of being classified as source locations, they are likely experiencing frequent reintroductions from elsewhere.

In the RGF model, areas of high cattle density were 2.6 times more likely to be classified as a branch-start (95% CI 1.26–5.52), and areas of low cattle density had a protective effect (OR 0.22 for branch-start, 95% CI 0.06–0.81) (Table 1). Thus, our inferred phylogeny indicated that FMDV branches tended to disperse from areas of high cattle density towards areas of lower cattle density. Given (1) the relatively short distances of branches examined in this dataset (median = 6.6 km) and (2) that the cattle density was assessed at the district level, it is somewhat surprising that this finding had statistical support. This implies between-district transitions occur with some regularity, and that cattle density could be used as an indicator of which neighboring districts are most at risk for introduction of the virus.

Using the RGF model, areas less than 14.8 km from livestock markets were 1.88 times more likely to be classified as a branch-start location (95% CI 1.05–3.39), indicating that these areas may act as a source of FMDV dissemination. Utilizing *seraphim*, we found that FMDV serotype O lineages in East Africa have a tendency to remain circulating near livestock markets [10]. Applying phylogeographic methods to FMDV serotype O in Ecuador, Carvalho et al. found that circulating strains had originated in areas of increased animal commerce [49]. In Cameroon, cattle trade is considered as the most significant risk factor for the dissemination of FMDV [50,51]. Livestock markets can be viewed as contact hubs, allowing for direct and indirect contact between animals from different areas and with different destinations [52]. Further, livestock traders and other stakeholders may unknowingly facilitate disease transmission as they move between markets [53]. Prioritizing market areas in prevention and control strategies may help to mitigate the spread of FMDV in endemic areas.

In this study, we have demonstrated the application of novel regression tools to time-stamped, georeferenced phylogenies. We chose to analyze only rasterized data for the RGF model presented here. However, non-rasterized data, such as management or seasonal factors, could also be explored in these models. Additionally, an advantage of both the SSF and RGF models is that they enable a multivariable approach, which is not currently possible with the suite of methods employed in the *seraphim* package. There are several reasons for why we chose to limit this analysis to our relatively small dataset of 48 FMDV serotype O sequences. A limitation of presence-only data, like that utilized in SSF models, is that the sampling bias has a stronger effect than in presence–absence models [54]. Thus, the implementation of SSF models for viruses should be reserved for situations in which sampling is reasonably representative of the distribution of cases, which we believe is the case for our Uganda dataset. This dataset was collected over two years, with 90% of the sequences collected in one year. In combination with a relatively small geographic area, this helped to reduce uncertainty in our inferred nodes. Finally, we chose to utilize the VP1 coding region of the FMDV genome because it is an important immunogenic site, and is therefore often the focus of FMDV molecular epidemiology studies [55,56,57,58,59]. It is possible that an analysis using whole genome sequences may have arrived at different results [60]. Additionally, the rainfall dataset utilized in this study is a 50-year mean, and thus does not capture any short-term changes in precipitation. Future models may aim to analyze the effects of seasonality or longer-term climate variations.

The methods presented here contribute to the growing field of landscape phylogeography by utilizing novel approaches to elucidate interactions between patterns of infectious disease spread and heterogeneities in environments. These tools could be used to develop risk-based FMDV control strategies following outbreaks. Instead of the somewhat haphazard “fire-brigade” approach to administering FMDV vaccinations, one might use these methods to determine which direction of spread is most likely given the attributes of the outbreak’s starting location. An understanding of FMDV source/sink locations may help veterinary officers to scale up or down their response to an outbreak when deciding how to best utilize limited resources. For example, an outbreak near a livestock market may warrant a more urgent and aggressive response than an outbreak far from a livestock market. These tools could be used to inform FMDV control strategies in other endemic settings, and, more broadly, for understanding the spread of any organism for which genetic sequences and corresponding spatiotemporal metadata exist.

## Figures and Tables

**Table 1:** Odds ratios (ORs) and corresponding 95% confidence intervals (CI) for the best-fitting resource gradient model. ORs represent odds of a node placed in the indicated category being classified as a branch-start location. The reference group for each covariate is the second tercile, i.e., the midrange values. Asterisks represent significant *p*-values (*p* < 0.05).

Variable	OR	95% CI	*p*-Value
Low rainfall	0.42	0.22–0.80	0.0008 *
High rainfall	0.66	0.32–1.34	0.253
Low cattle density	0.22	0.06–0.81	0.023 *
High cattle density	2.63	1.26–5.52	0.01 *
Near livestock market	1.88	1.05–3.39	0.034 *
Far from livestock mark	0.61	0.29–1.28	0.189

* denotes *p*-value < 0.05.

## Data Availability

See Velazquez-Salinas et al. (2020) and Supplementary Table S8 for FMDV sequences collected for this study.

## Supplementary Materials

The following are available online at https://www.mdpi.com/article/10.3390/pathogens11050524/s1; Supplemental Tables S1–S5 and S8; Supplemental Figures S6, S7 and S9.
