# Supplementary material for: Ecological and Anthropogenic Spatial Gradients Shape Patterns of Dispersal of Foot-and-Mouth Disease Virus in Uganda
Source: Pathogens. 2022 Apr 29;11(5):524. doi: 10.3390/pathogens11050524 (PMC9143568; doi:10.3390/pathogens11050524)

Supplemental Table S1. Marginal likelihoods of the path sampling/stepping-stone sampling under the molecular clock-population model combinations tested. The mean of the marginal likelihood of two Markov Chain Monte Carlo runs is shown. The best-fitting combination is highlighted.

	Molecular clock model			
	**Strict**		**Uncorrelated lognormal relaxed**	
Population model	path sampling	stepping stone sampling	path sampling	stepping stone sampling
constant	-2077.7891	-2077.80785	-2080.23665	-2080.802
exponential	-2085.69035	-2082.14985	-2081.09105	-2081.2094
GMRF Bayesian skyride	-2098.08835	-2098.2986	-2082.68895	-2082.8077
logistic	-2079.553	-2079.90305	-2081.3336	-2081.3028

Supplemental Table S2. Sources of covariate data.

**Environmental raster**	**Source**	**URL**
Mean annual rainfall	bioclim via R package *raster* v 3.1-5	www.worldclim.org
Cattle density	2008 National Livestock Census data (Ministry of Agriculture, Animal, Industry, and Fisheries); raster generated manually in AcGIS Pro (2.4.0)	https://www.ubos.org/wp-content/uploads/publications/05_2019THE_NATIONAL_LIVESTOCK_CENSUS_REPORT_2008.pdf
Human population density	Gridded Population of the World v4.11 2015	https://sedac.ciesin.columbia.edu/data/set/gpw-v4-population-density-rev11
Distance to nearest livestock market	Market locations: Famine Early Warning Systems Network; map generated, distances calculated using ArcGIS Pro	https://fews.net/east-africa/uganda/production-and-trade-flow-maps/january-2017-3
Distance to nearest major roadway	Roadway locations: The World Bank Data Catalog; distance raster generated using ArcGIS Pro	https://datacatalog.worldbank.org/dataset/uganda-roads
Distance to nearest international border	Shapefile: Humanitarian Data Exchange; distances calculated using ArcGIS Pro	https://data.world/ocha-rosea/6d6d1495-196b-49d0-86b9-dc9022cde8e7

Supplemental Table S3. Pearson correlation matrix for the predictors used in the step selection function (SSF) analysis.

**Environmental factor**	mean annual rainfall	cattle density	human population density	distance to livestock market	distance to roadway	distance to nearest international border
mean annual rainfall	1	0.032	0.26	-0.174	-0.328	-0.266
cattle density	0.032	1	0.423	-0.536	0.034	-0.517
human population density	0.26	0.423	1	-0.536	-0.405	-0.458
distance to livestock market	-0.174	-0.536	-0.536	1	0.335	0.347
distance to roadway	-0.328	0.034	-0.405	0.335	1	0.052
distance to nearest international border	-0.266	-0.517	-0.458	0.347	0.052	1

Supplemental Table S4. Cutoffs for the covariate classification used in the step selection function (SSF) and resource gradient function (RGF) models.

	**RSF model**		**RGF model**	
**Environmental factor**	1st tercile	3rd tercile	1st tercile	3rd tercile
mean annual rainfall (mm)	< 1197	> 1295	< 1187	> 1296
cattle density (cattle/square km)	< 67	> 119	< 67	> 123
human population density (people/square km)	< 113	> 544	< 105	> 654
distance to livestock market (km)	< 16.1	> 33.1	< 14.8	> 30.4
distance to nearest major roadway (km)	< 8.0	> 19.3	< 8.6	> 18.0
distance to nearest international border (km)	< 28.0	> 66.9	< 26.4	> 54

Supplemental Table S5. Pearson correlation matrix for the predictors used in the resource gradient function (RGF) analysis.

**Environmental factor**	mean annual rainfall	cattle density	human population density	distance to livestock market	distance to roadway	distance to nearest international border
mean annual rainfall	1	0.131	0.339	-0.253	-0.324	-0.294
cattle density	0.131	1	0.458	-0.513	-0.06	-0.51
human population density	0.339	0.458	1	-0.555	-0.387	-0.47
distance to livestock market	-0.253	-0.513	-0.555	1	0.328	0.373
distance to roadway	-0.324	-0.06	-0.387	0.328	1	-0.005
distance to nearest international border	-0.249	-0.51	-0.47	0.373	-0.005	1

Supplemental Figure S6. Location of Uganda (shaded blue) within the continent of Africa.

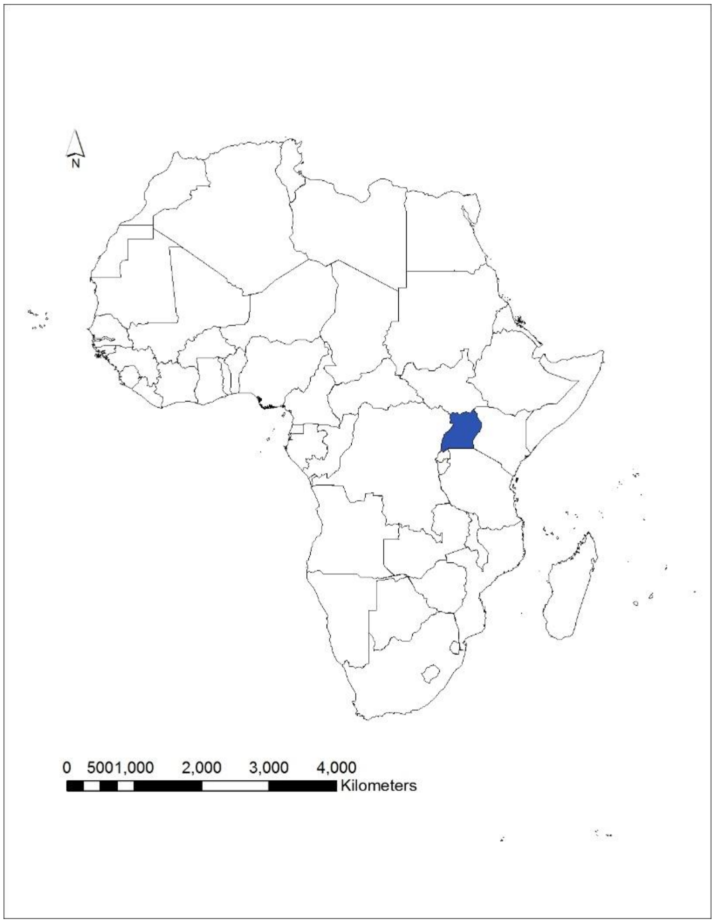

Supplemental Figure S7. A: Geographic locations of foot-and-mouth disease serotype O isolates used for this study. B: Reconstructed dispersal history of FMDV serotype O in Uganda: mapped maximum clade credibility (MCC) trees and 80\% highest posterior density (HPD) regions. MCC trees and HPD regions are based on 100 trees subsampled from the post burn-in posterior distribution. HPD regions were computed for successive time layers and then superimposed. HPD regions are colored according to their time of occurrence.

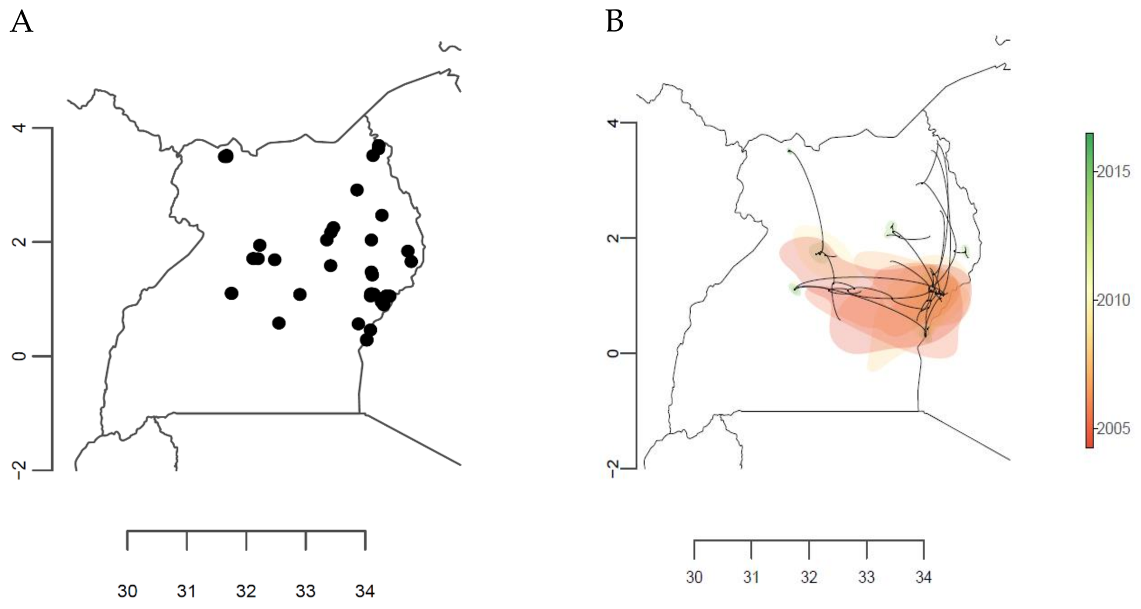

Supplemental Table S8. Sampling data for FMDV sequences.

Accession Number	Sampling Date	Latitude	Longitude
KY558284	2014-07-08	1.0885	34.145467
KY548406	2014-08-09	2.03641667	34.1043333
KY548409	2014-08-09	2.03766667	33.3539167
KY548411	2014-08-10	2.17041667	33.4232833
KY548412	2014-08-10	2.03766667	33.3539167
KY548428	2014-08-16	1.05336667	34.41425
KY548429	2014-08-16	1.05553333	34.3537167
KY558308	2014-08-17	0.95203333	34.2752167
KY558309	2014-08-17	0.9009	34.3114167
KY558311	2014-08-17	1.00591667	34.3336667
KY558310	2014-08-17	1.00618333	34.3341333
KY558314	2014-08-23	0.95801667	34.3051
KY558312	2014-08-24	1.84185	34.7179167
KY548438	2014-09-06	0.56743333	33.8851
KY548422	2014-09-13	1.0949	34.1104833
KY548424	2014-09-13	1.06496667	34.1070333
KY548413	2014-09-13	1.0569	34.0899333
KY548415	2014-09-13	1.05795	34.0936333
KY548417	2014-09-13	1.08711667	34.0944333
KY548418	2014-09-13	1.07273333	34.0925167
KY548419	2014-09-13	1.09271667	34.0939333
KY548425	2014-09-20	1.42395	34.12025
KY548427	2014-09-20	1.47571667	34.1037667
KY548478	2014-09-21	2.91326667	33.8626
KY558336	2014-10-16	1.65878333	34.7742667
KY558321	2014-10-18	1.58795	33.419
KY548462	2014-10-27	3.69193333	34.2273833
KY548464	2014-10-27	3.51606667	34.13115
KY548471	2014-10-28	3.63388333	34.2230833
KY558334	2014-11-14	2.46958333	34.2810167
KY548449	2015-01-06	0.28281667	34.02605
KY548443	2015-01-07	0.29245	34.0252333
KY548450	2015-01-07	0.28365	34.0262667
KY548453	2015-01-07	0.46048333	34.0883
KY558324	2015-01-07	1.10091667	31.7523
KY558325	2015-01-07	1.10091667	31.7523
KY558329	2015-01-07	1.10091667	31.7523

KY558330	2015-01-07	1.10433333	31.7492667
MH367347	2015-06-23	1.71116667	32.1275333
MH367344	2015-06-23	1.71155	32.1127833
MH367346	2015-06-23	1.70946667	32.1973
MH367342	2015-06-24	1.69023333	32.47722
MH367360	2015-07-02	1.94475	32.2265
MH367378	2015-09-17	0.58108333	32.5480333
MH367368	2015-11-28	3.49683333	31.63475
MH367377	2015-11-28	3.52005	31.6695667
MH367375	2015-11-29	3.49633333	31.6710333
KY548474	2016-07-02	1.08201667	32.9019667

Supplemental Figure S9. Inferred time-scaled phylogeny of FMDV serotype O VP1 sequences used for this study.

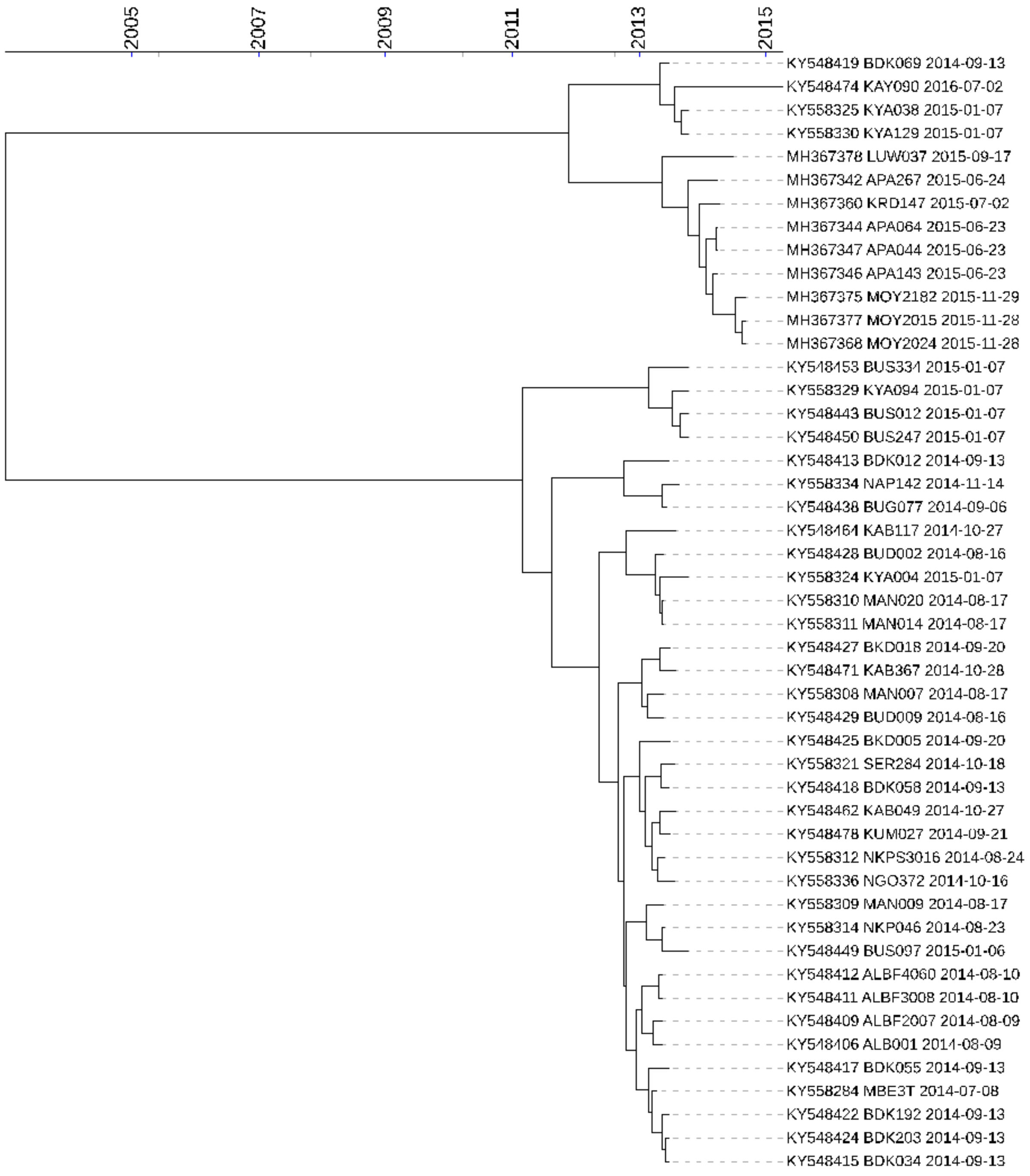

Supplement: Supplementary file 1 [file pathogens-11-00524-s001.zip › pathogens-1686077-supplementary.pdf]
